# Continuous Positive Airway Pressure in Elderly Patients with Severe COVID-19 Related Respiratory Failure

**DOI:** 10.3390/jcm11154454

**Published:** 2022-07-30

**Authors:** Elisa Ceriani, Annalisa Pitino, Dejan Radovanovic, Emanuele Salvi, Maddalena Matone, Thomas Teatini, Antonio Gidaro, Giovanni Tripepi, Pierachille Santus, Mercedes Gori, Chiara Cogliati

**Affiliations:** 1 Department of Internal Medicine, L. Sacco Hospital, Università Degli Studi di Milano, Via GB Grassi, 20157 Milano, Italy; emalele29@gmail.com (E.S.); matone.madda@gmail.com (M.M.); teatini.thomas@asst-fbf-sacco.it (T.T.); gidaro.antonio@asst-fbf-sacco.it (A.G.); chiara.cogliati@unimi.it (C.C.); 2 Institute of Clinical Physiology (IFC-CNR), Section of Rome, 00185 Rome, Italy; pitino@ifc.cnr.it (A.P.); mercedes.gori@ifc.cnr.it (M.G.); 3 Department of Pneumology, L. Sacco Hospital, Università Degli Studi di Milano, 20157 Milano, Italy; dejan.radovanovic@asst-fbf-sacco.it (D.R.); pierachille.santus@unimi.it (P.S.); 4 Institute of Clinical Physiology (IFC-CNR), Section of Reggio Calabria, 89124 Reggio Calabria, Italy; gtripepi@ifc.cnr.it

**Keywords:** continuous positive airway pressure, CPAP, ventilation, COVID-19, elderly

## Abstract

The elderly population represents a high percentage of patients hospitalized for COVID-19 pneumonia and severe respiratory failure, for whom CPAP may be a treatment option. The aim of this study was to describe the CPAP support modalities and to explore factors associated with CPAP failure. In this retrospective study, 110 consecutive patients aged ≥ 75 years were enrolled. Median frailty score, baseline partial arterial pressure of oxygen to fraction of inspired oxygen ratio (P/F), and respiratory rate (RR) were 5, 108, and 30 cycles/min, respectively. Of the 110 patients that began CPAP treatment, 17 patients died within 72 h from baseline, while in 2 patients, CPAP was withdrawn for clinical improvement. Thus, of the 91 patients still on CPAP at day 3, 67% of them needed continuous CPAP delivery. Patients with RR ≥ 30 and with frailty score ≥ 5 had an odds ratio of continuous CPAP needing of 3 and 4, respectively. Patients unable to tolerate CPAP-free periods demonstrated higher mortality risk as compared to those able to tolerate intermittent CPAP (OR: 6.04, 95% CI 2.38–16.46, *p* < 0.001). The overall in-hospital mortality was 63.6%. Delirium occurred in 59.1%, with a mortality rate in this subgroup of 83.1%. In a time-varying Cox model, the hazard ratio of death was 2.9 in patients with baseline RR ≥ 30 cycle/min, 2.4 in those with baseline P/F < 100. In the same model, the hazard ratio of death was 20 in patients with delirium and a frailty score < 5 and 8.8 in those without delirium and with frailty ≥ 5, indicating a competitive effect between these two variables on the death risk. **Conclusions:** Respiratory impairment, frailty, and delirium predict treatment failure, with the latter two factors demonstrating a competitive effect on mortality risk. CPAP support may represent a feasible therapeutic option in elderly patients, although chances of a therapeutic benefit are markedly reduced in case of severe respiratory impairment, very frail baseline condition or delirium occurrence.

## 1. Background

Severe acute hypoxemic respiratory failure (AHRF) is the most common complication of COVID-19 pneumonia and often necessitates admission to an intensive care unit for invasive mechanical ventilation with an associated mortality varying up to more than 50% [1,2]. In patients with AHRF, noninvasive respiratory support such as continuous positive airway pressure (CPAP), is widely used and has been implemented over time [3,4,5,6].

In more general terms, CPAP has been considered both as the first-line treatment for patients with moderate respiratory failure that may avoid tracheal intubation (OTI) [7,8,9] or the ceiling ventilatory care for patients not eligible for OTI for reasons of age and comorbidity [10].

Elderly patients are highly prevalent among patients hospitalized with COVID-19 pneumonia, suffering from milder forms that require hospitalization for the reason of a high frailty burden, to severe respiratory failure that requires an escalation to ventilatory support. In clinical practice, CPAP may often represent the ceiling ventilatory treatment, without robust indicators that may predict the failure of the respiratory support, particularly in these elderly patients [11].

Before COVID-19 experience, CPAP indications in the acute setting were pulmonary edema and eventually pneumonia [12,13]. In the community-acquired pneumonia setting, CPAP is commonly administered for few days [12], often allowing ventilation-free windows [13]. In COVID-19 pneumonia, AHRF course is typically longer, thus requiring prolonged respiratory support [14,15], with the most severe cases not able to tolerate short CPAP pauses. Considering these assumptions, the modality of administration and tolerability of CPAP support, particularly in elderly patients, may deserve further study. In fact, data about the use of CPAP support in the elderly population are still lacking, in terms of ventilator setting, modality of administration (intermittent vs. continuous), and patient tolerance, the latter of particular relevance considering the high risk of developing delirium during CPAP treatment [16]. Finally, an analysis of the factors impacting mortality in this high-risk population may be of relevance in order to improve clinical decision making, considering the risk of a futile approach and guide the communication with the patients and/or their relatives.

The aim of this study was to describe the characteristic of CPAP support and clinical outcomes in a cohort of patients ≥ 75 years old with COVID-19 pneumonia that underwent CPAP treatment. Moreover, we explored factors associated with CPAP failure, considering the frailty burden before the acute event, the severity of baseline respiratory impairment and the occurrence of delirium during treatment.

## 2. Methods

### 2.1. Study Setting and Population

This was a single center, retrospective, observational study conducted in the Internal Medicine and Respiratory Units of L. Sacco Hospital, Milan, Italy, one of the prominent tertiary care teaching hospitals involved as a COVID-19 hub during the current epidemic.

Elderly patients (age ≥ 75 years old [17]) hospitalized for AHRF due to COVID-19 pneumonia between 23 February 2020 and 14 April 2021 were consecutively enrolled. Patients were included if treated with helmet CPAP and if CPAP was considered as the ceiling respiratory support during hospitalization. The do-not-intubate order was made by the attending physician and the intensive care specialist, and based on patient’s clinical characteristics, including an extremely poor functional status prior to admission, very low probability of surviving the hospitalization, frailty score, and comorbidities, in addition to the patient’s own will when reliable [5]. This attitude was part of the clinical routine patients’ management and independent from study participation.

CPAP treatment, as part of local standard operating procedures, was started in patients receiving oxygen supplementation with a FiO_2_ of at least 0.50 through either Venturi or reservoir mask, with either clinical evidence of persistent respiratory distress (accessory muscle use or respiratory rate > 30 breaths per minute) or failure to achieve the target oxygen pulse oximetry (SpO_2_) > 94% in patients without chronic respiratory disease, or >90% in patients with prior diagnosis of chronic obstructive pulmonary disease (COPD), obstructive sleep apnea, or pulmonary fibrosis. CPAP was delivered through high-flow generators (VitalSigns Inc., Totowa, NJ, USA; 90–140 L·min^−1^; MYO 3133A, Pulmodyne) using a helmet (StarMed) with an adjustable PEEP valve (VitalSigns).

### 2.2. Data Collection

Accurate past medical history was obtained together with data about pre-existing morbidity; the comorbidity burden was also summarized with the Charlson Comorbidity Index. Performance status prior to hospitalization was evaluated by means of the Rockwood Frailty Scale [18]. A frailty score ≥ 5 was considered as a threshold to define significant frailty [19].

Vital signs, including respiratory rate (RR), arterial oxygen saturation measured by pulse oximetry (SpO_2_), and arterial blood gas (ABG) testing were collected before (within 6 h) the initiation of CPAP (Baseline parameters). The partial arterial pressure of oxygen to Fraction of inspired oxygen (P/F) ratio was calculated [20]. Among laboratory parameters collected at baseline (within 24 h before starting CPAP) the neutrophil to lymphocyte ratio (NLR) was registered.

CPAP parameters [FiO_2_ and positive end-expiratory pressure (PEEP)] were registered at baseline along with the overall treatment length. At baseline, the physician in charge of the patients administered CPAP treatment intermittently, allowing 1 h windows free from CPAP three times per day, if tolerated. During this CPAP-free window, oxygen was delivered by Venturi or reservoir mask. The subsequent eventual need of continuous CPAP treatment was otherwise established in the presence of SpO_2_ < 90% or the occurrence of respiratory distress when CPAP was removed, and oxygen was delivered by means of oxygen masks. After three days of treatment (T1), the type of CPAP demand (continuous or intermittent) was computed. Finally, data about outcome (CPAP failure intended as in-hospital death) and occurrence of delirium were registered. Delirium was defined as the occurrence of acute mental change from baseline combined with at least one of several key descriptors from hospital daily nurse/physician notes (e.g., delirium, confusional state, disorientation, hallucinations, agitation, inattention), accordingly to the chart-based delirium identification instrument [21]. Delirium was defined as a binary event (yes or no), and recurrent episodes were not pondered in our analyses. Finally, we registered the eventual administration of medications to improve patients’ CPAP tolerance and/or to treat delirium.

### 2.3. Study Endpoints

The endpoints of the study were: (1) to describe our elderly COVID-19 population managed with CPAP, in terms of CPAP treatment duration, ventilator parameters settings (PEEP and FiO_2_), and type of CPAP demand (continuous vs. intermittent CPAP at T1); (2) to identify baseline factors associated with CPAP demand (continuous vs. intermittent CPAP at T1); (3) to evaluate the mortality rate in the whole study sample and separately in patients with and without delirium; (4) to assess the relationship between baseline factors and delirium (as a time-varying covariate) and death; (5) to investigate, by an illness-death model, the risk factors for death in patients with and without delirium.

### 2.4. Statistical Analysis

Data were expressed as median and inter-quartile range or as absolute frequency and percentage, as appropriate. 

To assess the relationship between baseline characteristics and CPAP treatment demand (continuous vs. intermittent ventilation at T1), in patient that reached T1 time point univariate and multiple logistic regressions were performed. Tested covariates were age (as a continuous variable and as codified >80 vs. ≤80 years), gender (female vs. male), frailty score (as a continuous variable and as codified ≥5 vs. <5), RR (as a continuous variable and as codified ≥30 vs. <30 breaths/min, corresponding to the median value), P/F ratio (as a continuous variable and as codified <100 vs. ≥100), and NLR as a continuous variable. As a functional form of P/F ratio, RR, and frailty score, we adopted the binary one to comply with the general assumption underlying logistic regression. Because frailty score and Charlson Comorbidity Index are closely interrelated, these two variables were separately tested in multiple regression models. 

The survival analysis of time to delirium and time to death were performed through the Kaplan–Meier method in the entire population: the median survival times for delirium and death were calculated by the standard method, i.e., as the time corresponding to a cumulative survival of both outcomes of 50 percent.

The crude and adjusted effects of baseline covariates on the death risk were investigated by univariate and multiple Cox regressions. In these models, the effect of delirium was tested by considering this variable as time-varying covariate. In Cox models, data were expressed as hazard ratio, 95% confidence interval, and *p* value. The effect modification by frailty score (≥5 vs. <5) on the delirium–death link and vice-versa [i.e., the effect modification by delirium (yes vs. no) on the frailty score–death association] was investigated by simultaneously including frailty score, delirium, and their multiplicative term into the same Cox regression analysis. The hazard ratio (HR) of the frailty score by delirium strata in addition to the HR of delirium by frailty score strata were investigated by the linear combination method.

To investigate the predictors of delirium as intermediate variable in addition to the effect of the presence/absence of delirium on the relationship between a series of risk factors and death, an illness-death model was fitted. Illness-death models are a particular type of multistate models, wherein patients start the observation and subsequently may develop delirium, die straightforward, or die after achieving delirium.

In this model, the primary measures of interest are the pathway-specific hazard ratios. To model the effect of risk factors considering the intermediate endpoint (delirium), an illness-death model was fitted. In this analysis, three pathways were considered: pathway 1 going from baseline to death in patients with not delirium, pathway 2 going from baseline to delirium, and pathway 3 going from baseline to death after delirium. In this regression model, data were expressed as HR, with 95% confidence interval and *p* value.

Data were analyzed with STATA/IC 13.1 for Windows (College Station, TX, USA) and RStudio-1.2.5033.1.

The study (NCT04307459) was conducted according to the amended Declaration of Helsinki (2013). The study was approved by the local ethical committee (No. 17263/2020).

## 3. Results

### 3.1. Study Population

One hundred and ten patients were enrolled. Their median age was 81 years (IQR: 79–84); 66.4% were >80 years-old, and 79.9% were male. The most frequent comorbidities were hypertension (65.5%), cardiac disease (28.2%), and diabetes (24.5%). The Charlson Comorbidity Index (median value: 5, IQR: 4–7) and the frailty score (median value: 5, IQR: 4–5) were significantly interrelated (rho = 0.81, *p* < 0.001). Of the 110 patients that began CPAP treatment, 17 patients died before the T1 check point, while in 2 patients CPAP was withdrawn for clinical improvement. Thus, 91 patients were still in CPAP treatment at T1. Study population characteristics are reported in detail in Table 1. 

### 3.2. CPAP Treatment

All patients were affected by severe respiratory failure with a median P/F value before CPAP positioning of 108 (IQR: 81–170). CPAP treatment was started after a median time of 27 h from admission (IQR: 1–84) and delivered, at baseline, with a mean PEEP of 8.5 ± 2.7 cm H_2_O and a mean FiO_2_ of 0.62 ± 0.23. Respiratory parameters and laboratory variables at baseline are displayed in Table 1.

In the entire population, the median duration of CPAP treatment was one week (168 h, IQR: 72–288) or 230 h (IQR: 170–364) in patients who survived. Most patients (n = 91, 82.7%) were still on CPAP after 3 days from baseline (T1) and 67% of them were not able to tolerate ventilation-free windows at that time point, thus needing continuous support with CPAP (Table 1). 

Table 2 reports factors associated to the need for continuous CPAP at T1: in a multiple logistic regression model, including all variables associated with continuous CPAP at T1 with *p* < 0.10 at univariate analyses, the OR for continuous CPAP at T1 was 3.16 times higher in patients with RR ≥ 30 and 4 times higher in patients with a frailty score ≥ 5 than in those of the corresponding reference categories (Table 2). 

Patients unable to tolerate CPAP-free periods demonstrated a higher risk of mortality as compared to those able to tolerate intermittent CPAP at T1 (44/61 vs. 9/30 deaths, respectively, in patients in continuous vs. intermittent CPAP treatment, OR: 6.04, 95% CI 2.38–16.46, *p* < 0.001). Overall, in the 91 patients that reached the T1 point, the mortality rate was 58.2%. Among 19 patients that had CPAP treatment withdrawn before T1 (n = 19), in-hospital mortality was 89.5%.

### 3.3. Hospitalization Outcome and Prognostic Factors

The in-hospital mortality rate was 63.6%. Fifty percent of death occurred within one week from CPAP application (168 h IQR: 72–264). Overall, the median length of hospital stay was 14 days (IQR: 6–27): 8.5 days in patients with adverse prognosis (IQR: 4–15) and 32.5 days in survivors (IQR: 19.25–44). Among the 40 patients who survived, 27 were discharged home, while 13 were transferred to post-acute care facilities. 

During CPAP treatment, delirium occurred in 65 patients (59.1%). One half of them (33 patients) died within 6 days of its presentation (133 h, IQR: 47–301). The mortality rate was higher in patients that developed delirium than in those without (83.1% and 35.6% respectively; Chi2 *p* < 0.001). 

In 77.3% of patients, light sedation with opioids or antipsychotics was administered to improve tolerance to CPAP treatment and/or to manage delirium.

**Figure 1:**
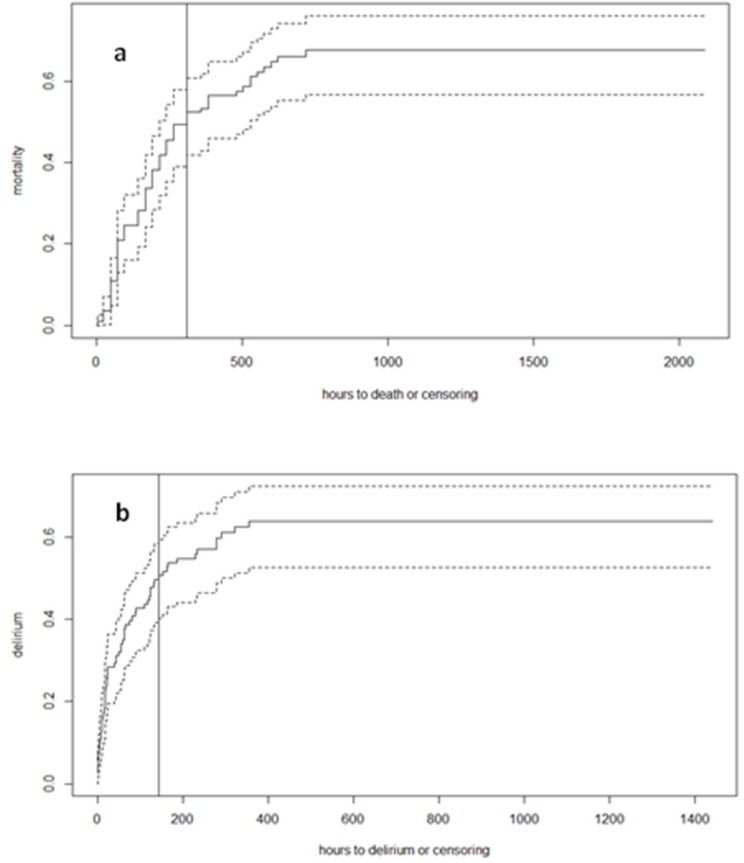
Time course of mortality and delirium onset. Panel (**a**). Time course of mortality (continuous line) and 95% CI (dotted lines). Panel (**b**) Time course of delirium onset (continuous line) and 95% CI (dotted lines). Full vertical lines represent the time point when 50% of mortality/delirium were reached.

In a Kaplan–Meier survival analysis (Figure 1), the median time to event was 2 weeks for mortality (Panel a, 312 h 95%, CI: 216–528) and about 1 week for delirium (Panel b, 143 h 95%, CI 88–291).

In univariate Cox regression analyses, the HRs of death increased with age, frailty score ≥ 5, RR ≥ 30 cycle/min, and P/F ratio < 100 (a) in Table 3. Notably, in a univariate Cox analysis testing delirium as a time-varying covariate, patients developing this complication had a HR of death 6.2 times higher than those without delirium. The median survival time in patients with delirium as time varying covariate was 4 days (96 h, 95% CI 72–192).

A multiple Cox model including delirium, frailty score, RR and P/F ratio at baseline (b) in Table 3 indicated that the HR for death was about 2.9 times higher in patients with RR ≥ 30 breaths/min and 2.4 times higher in patients with P/F ratio < 100 as compared to patients in the corresponding reference categories.

Remarkably, the effect of frailty score varies according to the presence/absence of delirium (b1 in Table 3) and, vice versa, the effect of delirium varies according to the presence/absence of a frailty score < 5 (b2 in Table 3).

In detail, the HR of death associated to a frailty score ≥ 5 was 8.83 (*p* = 0.01) in patients without delirium and 1.28 (*p* = 0.34) in those with delirium, and the two HRs significantly differed between them (effect modification, *p* = 0.03). Similarly, the HR of death associated to delirium was 20.23 in patients with frailty score < 5 (*p* < 0.001) and 2.92 (*p* < 0.001) in those without (effect modification, *p* = 0.03).

**Figure 2:**
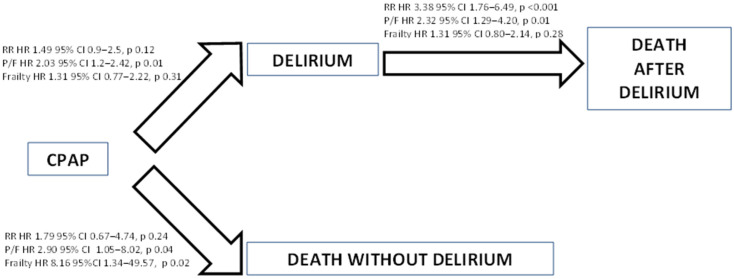
Illness-death model by baseline characteristics on transition to different states.

The illness-death model analysis (Figure 2) indicated that the HR to develop delirium was about two times higher in patients with P/F ratio < 100 than in those with P/F ratio ≥ 100 (*p* = 0.01), while frailty and respiratory rate HR on delirium occurrence risk did not reach statistical significance (frailty HR 1.31, *p* = 0.31; RR HR 1.49, *p* = 0.12). Once delirium occurred (see Figure 2 upper panel), the risk excess of post-delirium death associated to P/F ratio < 100 remained statistically significant (*p* = 0.01) in a model including RR (*p* < 0.001) and frailty score, the latter being not significant (*p* = 0.28).

Notably, in delirium-free patients (see Figure 2 lower panel), P/F ratio < 100 (*p* = 0.04) and frailty score ≥ 5 (*p* = 0.02) resulted to be associated to death whereas RR did not (*p* = 0.24).

## 4. Discussion

In our observational study, describing the clinical course of a cohort of elderly COVID-19 patients affected by severe respiratory failure treated with CPAP, overall mortality was 63.6%. We found that frailty, severity of respiratory failure, and delirium were independent predictors of death. Delirium occurred in 59.1% of patients, and 83.1% of them experienced adverse prognosis.

Older age is known to be associated with higher mortality risk in COVID-19 pneumonia patients [22]. Moreover, in elderly patients suffering for severe forms of respiratory failure needing high oxygen delivery, the mortality rate reaches 50–75% [23,24], in line with our data. In general, ‘do-not-intubate’ order patients with acute respiratory failure related to COVID-19 disease represent a high risk population even when considering slightly younger patients, as demonstrated by a recent meta-analysis, where the mortality rate among this subgroup was 72% [25].

A recent retrospective study conducted in patients not eligible to OTI raised even uncertainty about the superiority of CPAP treatment compared to standard oxygen therapy, although data must be considered with caution given absence of randomization and standardized parameters to initiate CPAP [26].

Our data demonstrated that frailty burden and baseline respiratory rate predict the need for continuously delivered CPAP support at day 3, which is associated with higher mortality risk. There are no available data about the type of CPAP demand (continuous vs. intermittent) and its association with prognosis in COVID-19 disease. Previous experience in interstitial lung disease related AHRF have indicated that the needing of continuous non-invasive ventilation support was associated with higher mortality risk in respect to those with non-continuous demand [27]. It is possible that the continuously delivered CPAP may play a detrimental role, as needing greater patient compliance and also implying issues in maintaining adequate caloric intake in frequently already compromised patients. Our data confirm the role of AHRF severity and Frailty on prognosis, aligned with previous studies that enrolled all ages groups [2,28].

Delirium was a very common complication in our population, occurring in about two in three patients, an incidence higher than what reported in previous studies evaluating frail and elderly patients with SARS-CoV-2 infection [29]. We may explain this result considering that we enrolled only patients who required CPAP treatment in reason of severe hypoxemia, which is a known predisposing factor for delirium [30], confirmed in our cohort where P/F ratio < 100 was significantly and independently associated with delirium occurrence. Moreover, helmet CPAP therapy itself predisposes to isolation and noise, other established predisposing factors for delirium [30].

In our population, the mortality rate in patients with delirium was 83.1%. The notion that delirium is associated with high mortality rates in hospitalized patients, particularly among elderly patients, is well established even for non-COVID cohorts [31], but in our population is higher than previously reported [16,32]. The difference can be explained by the coexistence of frailty and severe lung failure due to the acute illness.

Our data, indicating a competitive effect of delirium and frailty on mortality, provide elements to be considered when approaching such a complex patient; while in patients without delirium the basal frailty burden remains prognostic, the mortality risk once delirium has occurred is driven by delirium itself, and the major impact is exerted on less frail patients where it increases the HR of mortality by 20 times.

## 5. Limitations

It is not possible to infer from our study any definite conclusion on the role of CPAP in reducing mortality in reason of the retrospective design and of the absence of a comparator. However, the only available alternative treatment in the wards could have been standard oxygen therapy, which is unethical in reason of previous evidence of CPAP efficacy in COVID-19 disease [5,33,34]. The scarce availability of a high-flow nasal cannula oxygen delivery system or total face mask in our center have not allowed us to evaluate a comparison between treatments, potentially of particular interest in this elder and frail population where a more tolerable oxygen-support delivery modality could possibly have a positive effect.

## 6. Conclusions

We discovered that helmet CPAP treatment in a very frail and elderly cohort affected by severe COVID-19 disease demonstrated favorable outcome only in 36.4% of patients. The complexity of care was high, with two in three patients needing continuous CPAP after three days of treatment and delirium occurrence in a high percentage of patients.

Respiratory impairment degree, the pre-event frailty status, and delirium occurrence predict treatment failure, with the latter two factors indicating a competitive effect on mortality risk. Particularly, the occurrence of delirium was associated with a mortality higher than 80%. These observations, although needing confirmation in large multicenter studies, emphasize the complexity of management of older patients with severe respiratory failure: while CPAP support represents a chance of survival even in these patients, the risk of futile prolongation must be particularly considered in the presence of very frail baseline condition or occurrence of delirium.

## Figures and Tables

**Table 1:** Patients’ characteristics, laboratory findings and respiratory parameters at baseline according to the study outcomes.

Baseline Features of Study Population						
	Adverse Outcome			CPAP Demand at T1		
	Total (*n* = 110 *)	No (n = 40)	Yes (n = 70)	Total *(n* = 91 **)	Intermittent (n = 30)	Continuous (n = 61)
**Age (years) ^§^**	81 (79–84)	80 (78–81)	82 (79–86)	81 (78.0–84.0)	80 (79–81)	81 (78–85)
**Age > 80 years, *n* (%)**	73 (66%)	25 (65.5%)	48 (68.6)	57 (61.6%)	20 (66.7%)	37 (60.7)
**Male sex, *n* (%)**	87 (79.1%)	31 (77.5%)	56 (80%)	73 (80.2%)	23 (76.7%)	50 (82.0%)
**Hypertension, *n* (%)**	72 (65.5%)	25 (62.5%)	47 (67.1%)	58 (63.7%)	17 (56.6%)	41 (67.2%)
**COPD *n*, (%)**	7 (6.4%)	3 (7.5%)	4 (5.7%)	3 (3.3%)	0 (0%)	3 (4.9%)
**Chronic renal failure, *n* (%)**	15 (13.6%)	1 (2.5%)	14 (20%)	11 (12.1%)	4 (13.3%)	7 (11.5%)
**Diabetes mellitus, *n* (%)**	27 (24.5%)	9 (22.5%)	18 (25.7%)	20 (22.0%)	8 (26.6%)	12 (19.7%)
**CAD or heart failure, *n* (%)**	31 (28.2%)	6 (15%)	25 (35.7%)	21 (23.1%)	4 (13.3%)	17 (27.9%)
**Active cancer, *n* (%)**	7 (6.4%)	1 (2.5%)	6 (8.6%)	5 (5.5%)	1 (3.3%)	4 (6.5%)
**Pre-existing dementia, *n* (%)**	9 (8.2%)	0 (0%)	9 (12.9%)	5 (5.5%)	1 (3.3%)	4 (6.5%)
**Pre-existing psychiatric pathology, *n* (%)**	6 (5.5%)	4 (10%)	2 (2.9%)	4 (4.4%)	3 (10%)	1 (1.6%)
**Frailty Score ^§^**	5 (4–5)	4 (4–5)	5 (4–6)	5.0 (4.0–5.0)	4 (4–5)	4 (4–5)
**Frailty Score ≥ 5, *n* (%)**	65 (59%)	17 (42.5%)	48 (68.6%)	49 (53.8%)	10 (33.3%)	39 (63.9%)
**Charlson Comorbidity Index ^§^**	5.00 (4.00–7.00)	4.00 (4.00–5.00)	5.00 (4.00–7.25)	4.00 (4.00–6.00)	4.00 (4.00–5.00)	5.00 (4.00–6.00)
**Charlson Comorbidity Index ≥ 5, *n* (%)**	60 (54.5%)	15 (37.5%)	45 (64.3%)	44 (48.3%)	10 (33.3%)	34 (55.7%)
Respiratory parameters and Laboratory findings						
**Respiratory Rate (cycle/min) ^§^**	30 (25–35)	28 (23–32)	32 (28–36)	29.0 (24.0–32.0)	28 (24–31.5)	30 (25–36)
**Respiratory Rate ≥ 30 cycle/min, *n* (%)**	55 (50%)	13 (30.9%)	42 (60%)	44 (50.0%)	10 (33.3%)	34 (58.6%)
**P/F ^§^**	108 (81–170)	120 (100–194)	91 (76–145)	109.4 (82.8–170.0)	120.7 (99.1–185.5)	104.2 (78.7–153.5)
**P/F < 100, *n* (%)**	46 (41.8%)	8 (20%)	38 (54.3%)	37 (41.1%)	8 (26.7%)	29 (48.3%)
**NLR ^§^**	9.5 (5.4–14.2)	10.0 (5.5–14.7)	9.4 (4.9–14.4)	8.0 (5.15–13.0)	9.9 (7.5–12.9)	7.3 (4.9–12.7)
Treatment						
**Median CPAP treatment (hours)**	168 (72–288)	228 (168–348)	144 (71–216)	192 (144–312)	240 (168–438)	192 (120–264)

Legend: CAD: coronary artery disease; COPD: chronic obstructive pulmonary disease; RR: respiratory rate; P/F: partial arterial pressure of oxygen to fraction of inspired oxygen; NLR: neutrophil to lymphocyte ratio. * whole population enrolled in the study; ** patients on CPAP at T1 (after 3 days of CPAP treatment); ^§^ median value and interquartile range.

**Table 2:** Univariate and multiple logistic regression analyses by baseline characteristics. Dependent variable: type of CPAP demand at T1.

	Odds Ratio (Continuous vs. Intermittent)	95% CI	*p*
	Univariate model		
Age	1.06	0.9–1.2	0.32
Age (>80 vs. ≤80)	0.77	0.3–1.9	0.58
Gender (F vs. M)	0.72	0.25–2.2	0.55
* Frailty score (≥5 vs. <5)	3.5	1.4–8.9	0.007
RR (≥30 vs. <30 cycle/min)	2.83	1.1–7.1	0.027
P/F (<100 vs. ≥100)	2.6	1.0–6.7	0.052
NLR	0.99	0.9–1.0	0.81
	Multiple model		
RR (≥30 vs. <30 cycle/min)	3.16	1.15–8.70	0.026
* Frailty score (≥5 vs. <5)	4.08	1.48–11.22	0.006
P/F (<100 vs. ≥100)	2.67	0.94–7.55	0.065

* The inclusion into the model of the Charlson’s Comorbidity Index instead of the frailty score indicated that this latter had a predictive power for CPAP discontinuation that was of higher magnitude than that of the Charlson’s Comorbidity Index (OR: 2.28 95% CI: 0.88–6.13 *p* = 0.09). For this reason, we included the frailty score and not the Charlson’s Comorbidity Index into the model. Legend: RR: respiratory rate; P/F: partial arterial pressure of oxygen to fraction of inspired oxygen; NLR neutrophil to lymphocyte ratio.

**Table 3:** Univariate (panel a) and multiple (panel b) Cox analyses of death by baseline characteristics and delirium as a time-varying covariate.

		HR	95% CI	*p*
Univariate model (a)	Age	1.06	1.0–1.12	0.04 *
	Age (>80 vs. ≤80)	1.1	0.66–1.83	0.8
	Gender (Female vs. Male)	0.91	0.50–1.63	0.74
	* Frailty score (≥5 vs. <5)	1.88	1.13–3.11	0.01 *
	RR (≥30 vs. <30 cycle/min)	2.06	1.26–3.37	0.004 *
	P/F (<100 vs. ≥100)	2.73	1.67–4.44	<0.001 *
	GNL ratio	1.02	0.99–1.04	0.27
	Delirium ^#^ (yes vs. no)	6.17	3.47–10.96	<0.001
Multiple model (b)	Delirium ^#^ (yes vs. no)	20.23	4.21–97.07	<0.001
	Frailty (≥5 vs. <5)	8.83	1.71–45.64	0.01
	RR (≥30 vs. <30)	2.86	1.66–4.93	<0.001
	P/F (<100 vs. ≥100)	2.36	1.41–3.95	<0.001
	Delirium ^#^ (yes/no) by Frailty (≥5 vs. <5)	0.14	0.03–0.8	0.03
Effect modification of delirium (b1) ^§^	Delirium no			
	Frailty (≥5 vs. <5)	8.83	1.71–45.64	0.01
	Delirium yes			
	Frailty (≥5 vs. <5)	1.28	0.78–2.09	0.34
Effect modification of Frailty score (b2) ^§^	Frailty score < 5			
	Delirium ^#^ (yes vs. no)	20.23	4.21–97.07	<0.001
	Frailty score ≥ 5			
	Delirium ^#^ (yes vs. no)	2.92	1.56–5.47	<0.001

* The inclusion into the model of the Charlson’s Comorbidity Index instead of the frailty score indicated that the latter had a predictive power for CPAP discontinuation that was of higher magnitude than that of the Charlson’s Comorbidity Index (OR: 2.28 95% CI: 0.88–6.13 *p* = 0.09). For this reason, we included the frailty score and not the Charlson’s Comorbidity Index into the model. ^#^ Delirium was considered time-varying covariate. ^§^ Specification of interaction term of the multivariate model. Legend: RR: Respiratory Rate; P/F: Partial arterial pressure of oxygen to fraction of inspired oxygen; NLR neutrophil to lymphocyte ratio.
